# Editorial Expression of Concern: Glial β-Oxidation regulates *Drosophila* Energy Metabolism

**DOI:** 10.1038/s41598-020-70053-7

**Published:** 2020-07-31

**Authors:** Joachim G. Schulz, Antonio Laranjeira, Leen Van Huffel, Annette Gärtner, Sven Vilain, Jarl Bastianen, Paul P. Van Veldhoven, Carlos G. Dotti

**Affiliations:** 1 VIB Center for the Biology of Disease, Leuven and Center for Human Genetics, KU Leuven, Leuven, Belgium; 2 Laboratory of Lipid Biochemistry, KU Leuven, Herestraat 49, 3000 Leuven, Belgium; 3 Centro Biología Molecular “Severo Ochoa” CSIC-UAM, Madrid, Spain

Addendum to: *Scientific Reports*10.1038/srep07805, published online 15 January 2015

The Editors are issuing an Editorial Expression of Concern for this Article.

The Editors have been recently made aware by the Authors that neither the CPT2 fly stocks generated in this study nor DNA constructs with maps have been stored, and are therefore not available for request by readers wishing to repeat this work.

Joachim G. Schulz, Antonio Laranjeira, Leen Van Huffel, Annette Gärtner, Jarl Bastianen, Paul P. Van Veldhoven & Carlos G. Dotti agree with the Editorial Expression of Concern. Sven Vilain could not be reached.

